# Immunosenescence and Cytomegalovirus: where do we stand after a decade?

**DOI:** 10.1186/1742-4933-7-13

**Published:** 2010-09-07

**Authors:** Graham Pawelec, Arne Akbar, Peter Beverley, Calogero Caruso, Evelyna Derhovanessian, Tamas Fülöp, Paul Griffiths, Beatrix Grubeck-Loebenstein, Klaus Hamprecht, Gerhard Jahn, Florian Kern, Sven D Koch, Anis Larbi, Andrea B Maier, Derek Macallan, Paul Moss, Sandrine Samson, Jan Strindhall, Emanuelle Trannoy, Mark Wills

**Affiliations:** 1 Department of Internal Medicine II, University of Tübingen, Tübingen, Germany; 2 Division of Infection and Immunity, Department of Immunology, University College, London, UK; 3 Nuffield Dept. Medicine, University of Oxford, Oxford, UK; 4 Department of Pathobiology and Medical and Forensic Biotechnologies, University of Palermo, Palermo, Italy; 5 Research Center on Aging, Immunology Program, Geriatric Division, Faculty of Medicine, University of Sherbrooke, Sherbrooke, Canada; 6 Centre for Virology, Department of Infection, University College, London, UK; 7 Institute for Biomedical Aging Research, Austrian Academy of Sciences, Innsbruck, Austria; 8 Department of Virology, University of Tübingen, Tübingen, Germany; 9 Division of Medicine, Brighton and Sussex Medical School, Brighton, UK; 10 Department of Experimental Immunology, Academic Medical Center, Amsterdam, The Netherlands; 11 Department of Gerontology and Geriatrics, Leiden University Medical Center, Leiden, The Netherlands; 12 Centre for Infection, St George's, University of London, UK; 13 School of Cancer Sciences, University of Birmingham, Birmingham, UK; 14 Sanofi Pasteur MSD, Lyon, France; 15 Department of Natural Science and Biomedicine, Jönköping University, Jönköping, Sweden; 16 Sanofi-Pasteur, Lyon, France; 17 Department of Medicine, School of Clinical Medicine, University of Cambridge, Cambridge, UK

## Background and rationale

Since Looney at al. published their seminal paper a decade ago [1] it has become clear that many of the differences in T cell immunological parameters observed between young and old people are related to the age-associated increasing prevalence of infection with the persistent β-herpesvirus HHV-5 (Cytomegalovirus). Ten years later, studies suggest that hallmark age-associated changes in peripheral blood T cell subset distribution may not occur at all in people who are not infected with this virus [[2]; Derhovanessian et al., in press]. Whether the observed changes are actually caused by CMV is an open question, but very similar, rapid changes observed in uninfected patients receiving CMV-infected kidney grafts are consistent with a causative role [3]. This meeting intensively discussed these and other questions related to the impact of CMV on human immune status and its relevance for immune function in later life.

A more difficult question to answer is whether age-associated immune changes believed to contribute to the immunodeficiency of ageing are likewise caused by CMV. This seems *a priori *unlikely because CMV infection is very widespread, yet very elderly CMV-seropositive individuals are easily identified (although formally it is impossible to exclude that they were only recently infected). Thus, an important question becomes whether the CMV-sensitive altered immune cell subset distributions in the elderly, particularly the decreased numbers and percentages of naïve T cells and increased late-stage differentiated memory T cells (commonly used as biomarkers to assess "immunosenescence"), are in reality an epiphenomenon irrelevant for predicting health status in very old people. In order to discuss these and other relevant questions, a workshop was organised at the Center for Medical Research in Tübingen in December 2009, attended by virologists, immunologists and geriatricians whose brief was to elucidate the important questions on this issue, and make a first attempt at answering them. Questions focussed on the mechanisms of age-associated alterations to immunity, how far these alterations can be ascribed to CMV infection, and whether and to which degree other sources of "chronic antigenic stress" (eg. cancer, parasite infection, amyloid-β, other viruses, autoantigens) may be additive with CMV in this respect. The crucial question of how far we are able to assess the clinical impact of these factors using epidemiological approaches, longitudinal and cross-sectional studies was to be discussed, as well as the mechanisms responsible for such outcomes. The clinical relevance and predictive value of establishing immune signatures influenced by the above factors, in terms of longevity, cause of death, responsiveness to vaccination etc. must be established in order to make informed plans for translational work. Bearing this in mind, as well as the thus-far equivocal data on associations between mortality and CMV-seropositivity as a single factor, specifically, the questions which the participants tried to answer in this workshop were as discussed below. The number of important questions and paucity of anything approaching clear answers to most of them emphasizes the dire need for intensive investigations in this important area (Table 1).

**Table 1:** CMV and Immunosenescence: more questions than answers

	Innate immunity	Adaptive immunity			CMV	Clinical endpoints
		**CD4**	**CD8**	**B cells**		

Immunosenescence	altered	altered	altered	altered	present	?

IRP	?	altered	altered	altered	present	death

Frailty	?	?	?	?	present	disability

Vaccination	?	altered	altered	altered	present	?

### • What is immunosenescence?: how to define it:What are its immune signatures?

First, the exact meaning of the term "immunosenescence" was considered. A consensus view could be stated as follows: Immunosenescence is the impairment in immunity as a result of age-associated changes in function in a variety of cells: it is a phenomenon of decreased function, involving changes to both innate and adaptive immunity and a dysbalance between the two arms. Immunosenescence needs to be investigated not only in cells from the peripheral blood, but in tissues as well (easier said than done in humans). Any identified age-associated changes, if to be considered *senescence*, or "immune frailty", must be shown to contribute to deleterious clinical endpoints, such as decreased efficacy of vaccination in the elderly, for which there is some evidence (influenza, tuberculosis). This defect could stem from immune deficiency but also from defects at the site of vaccination (muscle) as well as cell recruitment (ie. in the interactions between aged tissues and components of immunity). A decreased ability to respond to pathogens in general is implied. All these points seem fairly obvious, but few have been well-investigated in appropriate studies. Hence, the general conclusion was that we need better markers for immunosenescence, and more data on its actual clinical relevance. There are many candidate markers but most of the correlations between such markers and any clinical endpoint remain largely hypothetical and in fact still require confirmation. Moreover, it was felt that many other factors, such as genetics, nutrition, stress, co-morbidities and socioeconomic factors are important in determining immunosenescence and clinical outcome, but that these have not yet been properly accounted for.

### • Relevance of the immune risk profile, IRP: general applicatibility? How do we measure it?

The concept of an "immune risk profile" (IRP) established from longitudinal studies of very elderly people in the southern Swedish city of Jönköping, did not investigate any correlations of immune parameters with response to vaccination. Instead, the IRP was defined solely in terms of mortality as a cluster of parameters including increased numbers of late-differentiated CD8 cells (but NOT decreased numbers of naïve CD8 cells) as well as low B cells: and CMV seropositivity [4,5]. The IRP was defined at baseline in 85 year-olds (note that even at 85 years, only ca. 15% of subjects are in the IRP group), with 2, 4 and 6 year follow-up. All people in the IRP group were CMV-seropositive, compared to around 85% in the non-IRP group.

The concept of the IRP is being applied to many different populations, but it is not clear whether this is appropriate. So far, as outlined above, we only know that it applies to very elderly Swedes from the town of Jönköping. Although there is *a priori *no real reason to suspect that other populations may differ, this is merely an assumption. Moreover, even in Sweden, we don't know at what age the IRP becomes relevant; very preliminary data suggest that it might start to be associated with excess mortality after age 65, but not at 55 [6]. Although longitudinal studies such as these are crucial, they remain rare, and practical considerations dictate that much of the available data in humans is and will remain derived from cross-sectional studies. It was therefore considered of great importance to pursue these types of longitudinal studies in diverse populations.

Whichever type of study is carried out, it was agreed that standardized multiparameter measurements are necessary, to be combined with factors not overtly immunological, ie. biological, functional and cognitive markers of ageing (including ADL, IADL, MMSE, GDS). If not using mortality as a robust but perhaps less than optimally informative endpoint, what clinical parameters can be utilised to assess the impact of immunosenescence? The above-mentioned functional tests could be good endpoints in elderly subjects. The assessment of the association between IRP and clinical frailty should be also considered as an endpoint. Skin testing (DTH reactions) would be a good endpoint, and is very different in young and old people. It is simple, ethical and cheap, but might reflect skin ageing more than immunosenescence directly. The response to vaccination would be another obvious end-point. However, it must also be borne in mind that recall responses can be less affected in the elderly, and that primary vaccination, eg. to a new strain of flu, may be difficult. For Zoster, whatever the humoural responses in the elderly, the reactivation of the virus is directly linked to decreased T-cell response levels that lead to the clinical problem. It was also suggested that simple in vitro functional studies might act to some extent as a surrogate, eg. the TNF response in whole blood to stimulation with LPS as a marker of general functionality (proliferation in the whole blood can be also considered). This should be easy to do, and is well-standardised.

#### How does CMV itself contribute to morbidity/mortality? What are the reactivation patterns of CMV, and does this occur more frequently in the elderly?? Is CMV reactivation an overlooked occult clinical problem at the end of life?

As discussed above, the presence or absence of CMV infection is one of the factors clustering with others to give the IRP in the very elderly. A major area for discussion at this workshop concerned the mechanisms responsible for this association. The main causes of death in the Swedish studies were infection and heart failure. One of the relatively less controversial findings regarding CMV and health status is the association with atherosclerosis and heart disease, where recent data have also indicated that not only CMV infection *per se *but the titer of anti-CMV antibody in the patient may correlate with survival time [7]. Thus, in all considerations of the impact of CMV on any measured parameter, it will be necessary to determine IgG antibody titers; it will also be informative to measure IgM, as well as antibody avidity and possibly specificity for the multiple CMV epitopes recognized by antibodies present in infected people. Given these provisos, and the near-ubiquity of CMV infection, it is not surprising that correlations between the mere fact of CMV infection and mortality are hard to establish.

An important question remains whether time of infection with the virus can be reflected in a more sophisticated serological analysis, as alluded to above. This is currently unknown. It is also unclear whether some individuals are simply more competent at controlling CMV and invest less resources in having to do so, with a possible ensuing advantage in later life. There is some preliminary data in familial longevity that this may be the case. The nature, profile or pattern of the CMV-response is likely to be more important than mere seropositivity. Patterns of anti-CMV antibody response in the young are very stable. Immediately after primary activation a full-blown response to every antigen is seen, which then decreases: IE1 is the first to decrease, pp150 persists over a longer time (IgM reactivity). Some IgM Abs persist for over 2 years but patterns can change during latency. For IgG, both specificity and quantity remain stable. Although IgG testing is very robust, there is no international standard for CMV Ab levels. All these aspects require attention.

CMV may also cause pathology by reactivation but we do not know how frequently CMV reactivates asymptomatically in the elderly, and if this is more frequent than in the young, whether it matters. Although geriatricians have a strong feeling that CMV end-organ disease is not a clinical problem in the elderly, by contrast with immunodeficiency syndromes where retinitis, or colitis may be clinically apparent, this might have been overlooked. So, for example, could bacterial pneumonia as a likely proximal cause of death actually follow occult CMV pneumonitis? Alternatively, the major impact of CMV might be indirect, perhaps by contributing to a low-level pro-inflammatory state, which is associated with frailty in the elderly [8].

Stress and inflammation also tend to activate CMV, but immunosuppression alone is not enough for reactivation; it seems that TNF is needed and NF-κB signalling is also important. So, will the low level pro-inflammatory status in the elderly lead to more reactivation? Is this potentially a vicious circle? Elderly people who become infected with the virus at old age from their grandchildren may well end up in the hospital with acute CMV infection, but this does not seem to happen otherwise. Hence, functional protection does appear to be well-maintained; CMV-specific immune responses are very heterogeneous and there is probably a great deal of redundancy in this crucial defence mechanism. It is believed that pp65 and IE1 are equally good in controlling CMV spread in transplant patients, but pp65-specific T-cells can sometimes be protective, sometimes not. There is also a notion that pp65 may be a decoy antigen, a way of evading the immune response of the host.

#### CMV and immune phenotype. What is the significance of the immune signature of CMV?

The biggest impact of CMV infection on immune parameters is to drive the accumulation of late-stage differentiated CD8+ T-cells, a prime characteristic of the Swedish IRP where increased absolute numbers and percentages of CD8+ CD27- CD28- cells contributed to the inverted CD4:8 ratio and poor proliferative responses, representing the major difference distinguishing the higher risk group from the rest of the population. CMV-specific CD8 clonal expansions have a low turn-over and fail to apoptose readily. However, whether these cells are deleterious or necessary for the host is unclear. Despite the concept of the finite immunological space, and the idea that these cells might "crowd out" others, there is little actual evidence that this does occur or, if it does, whether it is clinically relevant. Within these accumulated late-stage memory cell populations, repertoire narrowing might be important, besides proliferative senescence, apoptosis resistance and functional exhaustion (as implied by the very small and not-yet replicated study carried out on the Swedish sample, see ref. [9]). Such CD8 cells of different functional status (senescent-vs-non-senescent?) might be distinguishable by virtue of their expression of receptors like PD-1 or KLRG-1. However, each of these markers alone will probably not be sufficient to subdivide them, because, for example, KLRG1+ T-cells can have just as good a cloning efficiency as KLRG-1-negative cells if costimulated with 4-1BB. Probably no cell type is totally dysfunctional; this raises the question as to whether any is truly "senescent". It was concluded that this crucial question remains open.

Despite the widespread belief that decreased numbers of naïve cells in the elderly must be a bad thing, and the certainty that CMV infection drives down the numbers of such cells even further, whether this parameter is actually associated with a measured clinical outcome has not been properly tested in humans. One way of testing this would be to assess primary responses to neoantigens. Yellow fever vaccine may be a suitable example; however because it induces such strong responses, impairment in elderly cohorts may be difficult to measure. Less strongly stimulatory vaccines may be more revealing.

If CMV has such an over-riding effect on immune signatures, are there likely to be any additive effects of other viruses, pathogens or different sources of "chronic antigenic stress". There is some evidence that EBV has a minimal effect in addition to CMV, but that VZV and HSV do not. In other conditions such as rheumatoid arthritis, Alzheimer's Disease, and prostate cancer, there is some evidence consistent with CMV exacerbation; the same may be true of serious psychological stress. Evidence is sparse, but what is there suggests that there may indeed be additive immune "exhausting" effects of polypathologies.

#### Characteristics of immunosenescence and the IRP?

Many detailed questions were formulated in order to provide a framework for discussing the crucial gaps in knowledge concerning CMV and immunosenescence. These included asking whether immunosenescence starts at the level of the hematopoietic stem cell (HSC); the role of the thymus? Which biomarkers are best? What impact does CMV have on the HSC microenvironment, thymus? Is there no age-associated decrease of CD8 naïve cells in CMV-negative people? Is the decrease in naïve cells in CMV+ people limited to CD8, and not CD4, cells? Is the decrease of naïve cells associated with any clinical outcome? Are T-regs altered with age and what is the effect of CMV thereon? Does CMV affect B cells (part of the IRP)? Is there a contribution of innate immunity to the IRP? What is the impact of CMV on cells bridging innate and adaptive immunity (Th17, Tγδ, NK, NKT, DC)? What is the relevance of telomere length measurements? Relevance of humoural factors, cytokines, such as type I IFN blocking telomerase; CMV induces type I IFN, and feedback will have to be established. Systems approaches will need to be invoked for a full understanding of these interactions.

Regarding the impact of CMV on HSC, it now seems clear that most cell types can be infected, not just myeloid and endothelial cells. Although immediate early and late genes are not expressed in CD34+ HSC, two proteins are candidates for latency-associated viral products, and RNA has been detected in stem cells. However, only a small proportion of stem cells seems to be infected, so the effect of CMV on HSC is probably negligible. On exiting the bone marrow, T cell progenitors must be processed in the thymus. Despite the well-known but nonetheless still little-understood phenomenon of age-associated thymic involution, even the very elderly commonly seem to retain some residual thymic function. Is thymic output different in CMV+ and CMV- individuals? This was felt to be an important question to answer, too. Given the difficulty in identifying naïve cells in the elderly, this may not be so easy as would appear at first sight. How should we define naïve cells? Telomere lengths and TRECs are probably the best way, but patterns of cytokine production and surface markers CD31, LFA1 should be assessed as well.

Another area of great topical interest concerns Tregs: their frequency appears to increase with age but the impact of CMV on these cells is only just beginning to be studied. The frequency of Foxp3 expression appears similar in clonal expansions of CMV-specific T-cells and total CD4+ T-cells and the frequency of Tregs is similar in CMV+ and CMV- individuals. Of course, more subtle changes in Treg subpopulations or functional changes may be significant. Do Tregs make the virus reactivate more or less? Similarly, are there any CMV-specific Th17 cells and do they differ between young and old?

There was also much discussion on components of the innate immune system, CMV and immunosenescence. It was noted that γδ-T cells expand after CMV infection in transplant models, but very little has been described concerning these cells in ageing, and the impact of CMV. Similarly, although of great importance in CMV immunosurveillance, and probably also in immunosenescence, the impact of NK cell status on morbidity/mortality in the elderly is very much an open question. It was noted that low NK cytotoxicity may predict cancer recurrence, as well as infectious disease susceptibility in later life, but, again, data are very scarce. NKT cells are also likely to play some role in this respect. There may be enrichment of a certain type of NK cell accumulating in CMV+ individuals, and these also grow out of peripheral blood cells when cocultured with CMV-infected fibroblasts. Some rather subtle changes to DC and antigen presentation have also been noted in ageing, and antigen presentation is known to be decreased in CMV+ individuals. This also needs clarification in the context of immunosenescence. Neutrophils can also be infected by CMV and their role in chronic viral infections, especially reactivation, also requires attention.

#### Could there be an early-life or any other benefit from being infected with CMV?

Finally, it was considered whether there is any evidence for beneficial effects of CMV, helping to explain its unique interactions with immunity and the inability of the host to eliminate the virus? For example, cells expanded in CMV+ patients may protect against cancer, as there is some evidence for lower cancer rates in CMV+ individuals. Other advantages from CMV-infection might be reduced allergies or infections in early life, due to the increased proinflammatory status of infected people. Less alloreactivity and hence organ graft rejection may be seen in patients with high levels of some CMV-specific T cells [10].

### • Interventions? Should we try to suppress CMV?

The overall feeling was that CMV is bad for you. On balance, its elimination was thought to be desirable. Vaccination is one option, and the first report recently of a vaccine for preventing acquisition of primary infection by seronegative mothers is encouraging [11]. Therapeutically, vaccination in CMV+ individuals might be able to boost antibody responses. However the logic of this approach depends on inducing a different type or scale of immune response to that induced by natural infection, which we know does not achieve clearance of CMV from infected individuals, and not from lack of magnitude. Vaccine trials are ongoing. In the case of VZV therapeutic vaccination, 50-60% reduction of Zoster incidence in the treated group has been attained, making it conceivable that something similar might be achieved for CMV. Other approaches are certainly possible, but the question remains: if we get rid of CMV what will be the impact?

At the end of this First International Workshop on CMV & Immunosenescence, the attendees decided that it would be extremely valuable to repeat the exercise in a year's time in order to update participants on progress, include areas and experts omitted from or unable to attend the first workshop and identify new areas of investigation. To this end, the Second International Workshop on CMV & Immunosenescence will be held in Cambridge, UK, 2-4^th ^December, 2010, organised by Mark Wills.

## Competing interests

The authors declare that they have no competing interests.

## Authors' contributions

All authors attended the Workshop, participated in the discussion, saw and commented on the text published here. All authors read and approved the final manuscript.

## Note

Report from the First International Workshop on CMV and Immunosenescence held in Tübingen, Germany, 15-17 December, 2009
